# Interleukin-7 Resensitizes Non-Small-Cell Lung Cancer to Cisplatin via Inhibition of ABCG2

**DOI:** 10.1155/2019/7241418

**Published:** 2019-12-14

**Authors:** Bin Ke, Ting Wei, Yuanyuan Huang, Yuxin Gong, Gang Wu, Junfang Liu, Xiaoting Chen, Lin Shi

**Affiliations:** ^1^Department of Traditional Chinese Medicine, The First Affiliated Hospital of Sun Yat-sen University, China; ^2^Department of Cancer Center, Zhujiang Hospital of Southern Medical University, China; ^3^Department of VIP Ward, Affiliated Cancer Hospital of Sun Yat-Sen University, China; ^4^Department of Respiratory Diseases, Zhujiang Hospital of Southern Medical University, China; ^5^Department of Traditional Chinese Medicine, Zhujiang Hospital of Southern Medical University, China

## Abstract

Treatment with cisplatin (DDP) is one of the standard therapies used to treat non-small-cell lung cancer (NSCLC) and fundamentally causes resistance in cancer cells, which eventually poses as an obstacle to the efficacy of chemotherapy in NSCLC. Efforts are on all over the world to explore a sensitizer of NSCLC to DDP. Here, we studied the effect of IL-7 on the resistance of NSCLC to chemotherapy. We observed that IL-7 treatment significantly enhanced DDP-induced effects in A549 and A549/DDP cells (DDP-resistant cells), including decreased cell viability and proliferation, as well as increased cell apoptosis and S arrest, indicating that IL-7 treatment resensitized DDP-resistant NSCLC cells to DDP. Subsequently, IL-7 enhanced the sensitivity of PI3K/AKT signaling and expressions of ABCG2 to DDP. By inhibiting IL-7 signaling via IL-7R knockdown or activating PI3K/AKT signaling via PI3K activation, the resensitization to DDP by IL-7 was abrogated, and the expression levels of ABCG2, p-PI3K, and p-AKT were found to be significantly higher. In vivo results also confirmed that IL-7 only in combination with DDP could remarkably induce tumor regression with reduced levels of ABCG2 in tumorous tissues. These findings indicate that IL-7, apart from its adjuvant effect, could overcome multidrug resistance of DDP to restore its chemotherapy sensitivity.

## 1. Introduction

Lung cancer is one of the most commonly diagnosed cancers and the leading cause of cancer-related deaths worldwide, and approximately 85% of all cases of lung cancer are characterized as non-small-cell lung cancer (NSCLC). Cisplatin (DDP) is the most frequently prescribed drug for various cancers, with nearly 50% NSCLC patients being estimated to receive treatment with DDP [1]. It has been shown through a large number of studies that cancer cell apoptosis resulting from DNA lesions by DDP exposure is the most acceptable mechanism underlying its anticancer effect [2]. Unfortunately, resistance to DDP therapy is always formed likely to other types of chemoradiotherapy, resulting in 5-year survival of less than 25% and local disease failure in up to 50% of these patients [3]. Therefore, efforts to investigate DDP sensitizers, improve NSLCL control, and prolong survival are on.

Solid reports have demonstrated recognizable contributions by immune response to anticancer and have shown that the dysregulation of the immune system by chemotherapy has been reported by many emerging studies to contribute significantly to the defect of immune surveillance, resulting therapy resistance, cancer development, and progression [4, 5]. Immune-related agents are increasingly being used only in combination with other drugs to promote sensitization of cancers. Interleukin-7 (IL-7), a classic immune cytokine, mainly produced by epithelial and stromal cells, controls T cell proliferation and survival [6, 7]. IL-7 has been shown to be associated with the development of cancers in some studies. A study has recently reported that IL-7 contributes significantly to the invasion and migration of prostate cancer cells [8]. IL-7 appears to promote bladder cancer cell proliferation according to Park et al. [9]. However, IL-7 has inhibitory effects on a variety of cancers, including glioma, melanoma, lymphoma, leukemia, and glioblastoma [10]. It has also been shown that intratumoral IL-7 injection transduced dendritic cells resulting in complete tumor regression in a murine lung cancer; IL-7 administration increased sensitization of metastatic nodules to radiofrequency thermal ablation in lungs [11, 12]. However, the role of IL-7 in resensitization-resistant NSCLC to DDP remains elusive.

Aberrant influx and efflux of drugs play an important role in acquired resistance of cancer cells to a variety of chemotherapies. A member of the ATP-binding cassette (ABC) transporter family, ABCG2 (BCRP1) is an important participant in drug influx and efflux, and its overexpression predicts the poor outcome of chemotherapy [13, 14]. DDP treatment has been reported in a few studies to induce the expression of ABCG2, which in turn confers the resistance of tumors cells to DDP, including ovarian cancer and NSCLC [15, 16]. Inhibition of ABCG2 by miR-495 also has been found to reverse DDP resistance in the relevant resistant NSCLC cells [17].

This is the first report indicating that IL-7 resensitized NSCLC to DDP in vitro and in vivo. It was also observed that the resensitization might be derived from the regulation of IL-7 in the expression of ABCG2.

## 2. Materials and Methods

### 2.1. Cell Culture

The lung cancer cell line A549 and its cisplatin- (DDP-) resistant cell line A549/DDP acquired from the American Type Culture Collection (ATCC) were used in this study and maintained using Dulbecco's modified Eagle's medium (DMEM) containing 10% FBS (Life Technologies, Grand Island, NY, USA) and ampicillin and streptomycin at 37°C and 5% CO_2_.

### 2.2. Cell Transfection

IL-7R alpha siRNA was used to knock down IL-7R expression in the cells. Briefly, GenePharma (Shanghai, China) supplied the IL-7R alpha siRNA (si IL-7R*α*) and the negative control (NC) which were transfected into the A549/DDP cells using Lipofectamine 2000 (Invitrogen Carlsbad, California, USA), according to the manufacturer's instructions. The sequences of the siRNAs were as follows: IL-7R alpha siRNA: 5′-GUCAGUAACUCUACUUGCU-3′, and NC: 5′-UUCUCCGAACGUGUCACUU-3′.

### 2.3. Tumor Model

Female nude BALB/c mice, aged 4–5 weeks, weighing 16–18 g, were procured from Southern Medical University and lodged in an SPF room under controlled conditions, including a 12 h light-dark cycle, a temperature of 23 ± 3°C, and a relative humidity of 55 ± 15%. The Institutional Animal Care and Use Committee of Zhujiang Hospital of the Southern Medical University approved by the animal protocol.

The xenograft models of human lung cancer DDP-resistant cell line A549/DDP was established by subcutaneously injecting the cells (4 × 10^6^) into the mice. When tumors were palpable, the mice were randomly divided into 4 groups (*n* = 8) and treated with 0.1 mL/10 g twice a week intraperitoneally with a control vehicle (PBS), IL-7 (2 *μ*g/injection; eBioScience, San Diego, CA, USA), DDP (5 mg/kg; Sigma, St. Louis, MO, USA), and IL-7 and DDP as described hereinbefore. The tumor sizes were measured once in three days apart and the tumor volumes examined weekly once and were calculated as *V* (cm^3^) = width^2^ (cm^2^) × length (cm)/2.

### 2.4. CCK-8 Assay

Viability of the cells was assessed using Cell Counting Kit-8 (CCK-8; Dojindo, Kumamoto, Japan). A549 and A549/DDP (5 × 10^3^) cells were seeded into each well of a 96-well culture plate each. The A549 and A549/DDP cell lines were treated with IL-7, DDP, or their combination as indicated doses for 24 h, 48 h, and 72 h. Subsequently, the cells were harvested and washed with PBS, cultured in 10% DMEM, incubated for 2 h at 37°C, and the absorbance measured at 450 nm by a microplate reader.

### 2.5. EdU Assay

Posttreatment, the A549/DDP cells were labeled with 100 *μ*L of 50 *μ*M 5-ethynyl-2′-deoxyuridine (EdU; Ruibo Biotech, C10327, Guangzhou, China) for 2 h. They were thereafter detected using Apollo® 643; cell nuclei were counterstained with DAPI (BioWord, Minnesota, NY, USA). The EdU-stained cells were observed using a fluorescent microscope (AMG EVOS; FL, USA).

### 2.6. Colony Formation Assay

The A549/DDP cells were collected and resuspended in a complete 10% FBS medium. After being seeded into 12-well plates, the cells were treated as described for a period of 48 h. Subsequently, the cells were fixed using methanol for 15 min and stained with 0.1% crystal violet (Beyotime, Shanghai, China). An inverted light microscope (Olympus, Tokyo, Japan) was used to observe the colonies, and the surviving colonies of >50 cells were scored.

### 2.7. Determination of Cell Apoptosis and Cycle In Vitro and In Vivo

Hoechst assay and flow cytometry were used for cell apoptosis analysis in vitro. For the Hoechst assay, A549/DDP cells were collected, fixed using 4% PFA, stained with 0.1 *μ*g/mL Hoechst 33342 (Sigma, St Louis, MO, USA), and then examined using a fluorescence microscopy with a filter for Hoechst 33342 (365 nm). For the flow cytometry assay, the harvested cells were suspended in tubes and incubated in a solution of 2 *μ*L each of Annexin V and Propidium Iodide (PI; eBioscience, San Diego, CA, USA) for 30 min as per the manufacturer's instructions. An FACSCalibur instrument was used to analyze the cells. Cells were stained with PI staining solution (10 *μ*g/mL RNase A and 50 *μ*g/mL PI) at 37°C for 30 min in the dark to study the cell cycle. The cell cycle distribution was also analyzed using flow cytometry with the help of the CellQuest software.

Apoptosis analysis in vivo was performed using a terminal deoxynucleotidyl transferase-mediated dUTP-biotin nick end labeling (TUNEL) assay. Tumor tissue sections were stained with TUNEL, using an apoptosis in situ detection kit (Wako Pure Chemical, Osaka, Japan), as per the instruction. Brown stained cells were apoptotic under light microscopy.

### 2.8. Preparation of Cell Extracts and Western Blotting

The A549/DDP cell line or tumor tissues were rinsed with PBS and lysed in a RIPA buffer with protease inhibitors and phosphatase inhibitors (Roche Applied Science, Indianapolis, IN, USA) for Western blots. The proteins were segregated from tumor samples or cell lines. Procedures for immunoblotting are described elsewhere [18]. Primary antibodies against IL-7 (Abcam, ab9732, 1 : 3000 dilution), IL-7R (Abcam, ab180521, 1 : 1000 dilution), PI3K (CST, 13666, 1 : 1000 dilution), p-PI3K p85 alpha (phospho Y607) (Abcam, ab182651, 1 : 1000 dilution), AKT (CST, 4691, 1 : 1000 dilution), p-AKT (CST, 4691, 1 : 2000 dilution), ABCG2 (Abcam, ab130244, 1 : 1000 dilution), and GAPDH (Abcam, ab181602, 1 : 10000 dilution), as well as HRP-conjugated goat anti-rabbit secondary antibodies (Abcam, ab7090, 1 : 10000 dilution) and HRP-conjugated goat anti-mouse secondary antibodies (Abcam, ab47827, 1 : 5000 dilution), were procured from Abcam (Cambridge, MA, USA) or Cell Signaling Technology (Denver, MA).

### 2.9. Immunofluorescence

Tumor cells or tissues were fixed with 4% formaldehyde in PBS for 15 min and rinsed with PBS thrice. The cells or tissues were permeabilized using 100% methanol for 10 min at −20°C and blocked with 3% bovine serum albumin (BSA) in PBS for 60 min and incubated with primary antibodies against ABCG2 and p-AKT overnight at 4°C. The coverslips were rinsed thrice in PBS and were incubated in FITC-conjugated IgG (Santa Cruz Biotechnology, sc-2359, USA) or PE-conjugated IgG (Santa Cruz Biotechnology, sc-3753, USA) for 1–2 h at room temperature in the dark, and then the nucleus was stained with DAPI (BioWord, Minnesota, NY, USA). They were then observed using a FV10i confocal microscope (OLYMPUS, Japan).

### 2.10. Immunohistochemistry

The Ki-67 expression in tumor tissues was assessed on 2 *μ*m thick, formalin-fixed, and paraffin-embedded specimen sections immunohistochemically. Slides were cleared of the wax, antigen unmasked, and then endogenous peroxidase activity blocked using 3% hydrogen peroxide for 10 min at room temperature and rinsed. Anti-Ki-67 antibody (Abcam, ab15580) was used to incubate the FFPE specimen sections overnight at 4°C and the EnVision Detection System kit (DAKO, Denmark) for the DAB chromogen followed by nuclear staining with hematoxylin.

### 2.11. Statistical Analyses

All data are presented as mean ± SD. Statistical analysis was carried out using Prism (GraphPad Software). The two groups were compared using unpaired *t*-tests, and multiple groups were compared with one-way ANOVA. *p* values < 0.05 were considered statistically significant.

## 3. Results

### 3.1. IL-7 Restored the DDP Sensitivity in A549/DDP Cells

**Figure 1:**
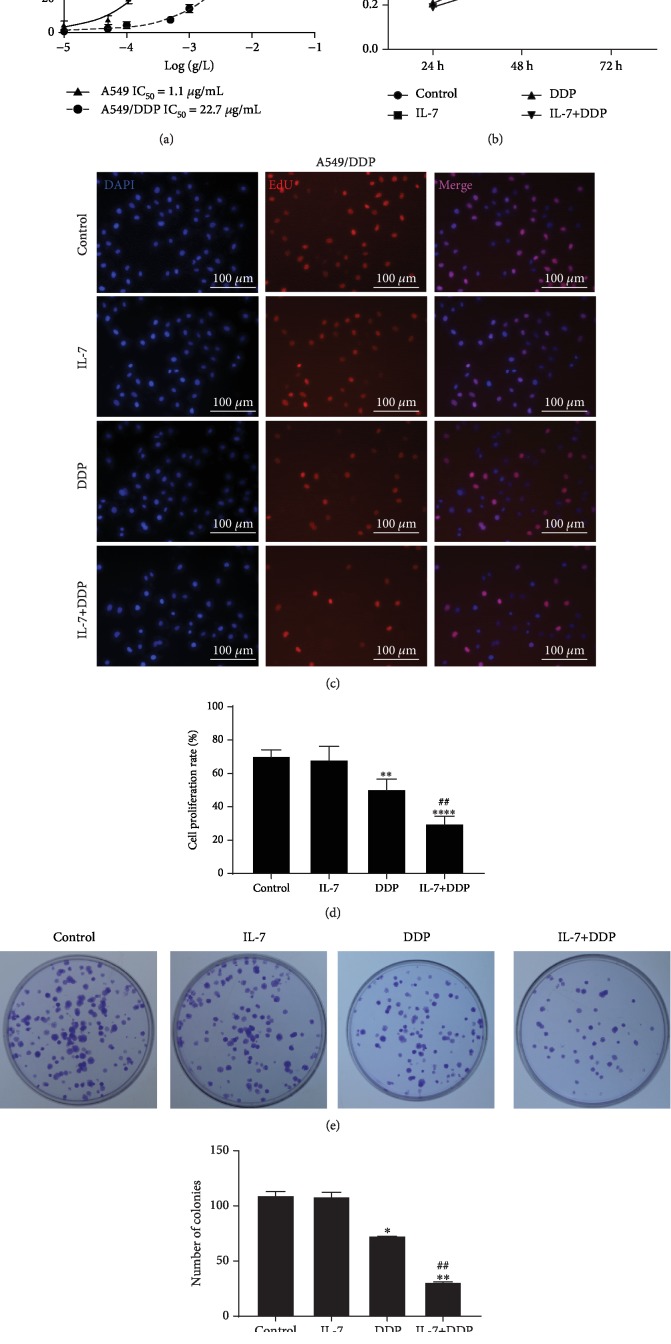
The addition of IL-7 sensitized the A549/DDP cells to DDP-induced tumor inhibition. (a) A series of DDP (0–50 *μ*g/mL) doses was added to the human lung cancer cell line A549 and DDP-resistant cell line A549/DDP. The cell vitality was estimated by CCK-8 assay and the IC_50_ value (half-maximal (50%) inhibitory concentration) was calculated. (b) A549/DDP cell lines were treated with IL-7 (2 ng/mL) and/or DDP (0.5 *μ*g/mL) for 24 h, 48 h, and 72 h; the cell vitalities were analyzed by CCK-8 assay. (c, d) EdU staining was performed 48 h after the indicated treatment to estimate the proliferation of the A549/DDP cell line. (e, f) Colony formation assay and the quantified number of colonies of A549/DDP cells after the indicated treatment. ^∗^*p* < 0.05, ^∗∗^*p* < 0.01, and ^∗∗∗∗^*p* < 0.0001, as compared with the control group; ^#^*p* < 0.05 and ^##^*p* < 0.01, as compared with the DDP group. Data are presented in terms of mean ± SD.

The effect of IL-7 on chemotherapy resistance of NSCLC was studied using two lung cancer cell lines, A549 and its corresponding DDP-resistant cell line A549/DDP. A series of DDP doses (0–50 *μ*g/mL) was added to the two cell lines for 24 h, and A549/DDP cells were found more resistant to DDP than A549 cells with higher cell vitality and significantly higher IC_50_ value (half-maximal (50%) inhibitory concentration) (Figure 1(a)). A549/DDP cells showed no particular cytotoxicity when treated with 0.5 *μ*g/mL DDP, whereas A549 cells exhibited significant inhibition, owing to which this dose was used in the following experiments. A series of IL-7 doses (0–8 ng/mL) was added to the two cell lines for 24 h; the viability of A549 or A549/DDP cells was comparable with that of the control. Results indicated no cytotoxic effect of IL-7 (2 ng/mL) on both the cell lines (Supplementary [Supplementary-material supplementary-material-1]). Thus, this dose of IL-7 was used in the following investigations. After exposure to IL-7 at 2 ng/mL for 24 h, 48 h, and 72 h, the viabilities of A549/DDP cells were compared with those of the control (Figure 1(b)), indicating no cytotoxic effect of this dose of IL-7 on the DDP-resistant cells. Nevertheless, DDP in combination with IL-7 was observed to inhibit cell viability significantly when compared with the DDP treatment alone, demonstrating a synergetic effect of IL-7 on DDP-induced cytotoxicity in the resistant cells. The combination of IL-7 with DDP significantly enhanced the sensitivity of A549/DDP cells with lower proliferative activity as shown by the EdU assay (Figures 1(c) and 1(d)), also confirmed by the colony formation assay (Figures 1(e) and 1(f)). Thus, IL-7 can be said to recover the inhibitory effect of DDP on proliferation in the DDP-resistant cell line A549/DDP.

### 3.2. IL-7-Enhanced DDP-Induced Apoptosis and Cell Cycle Arrest in Resistant Cells

**Figure 2:**
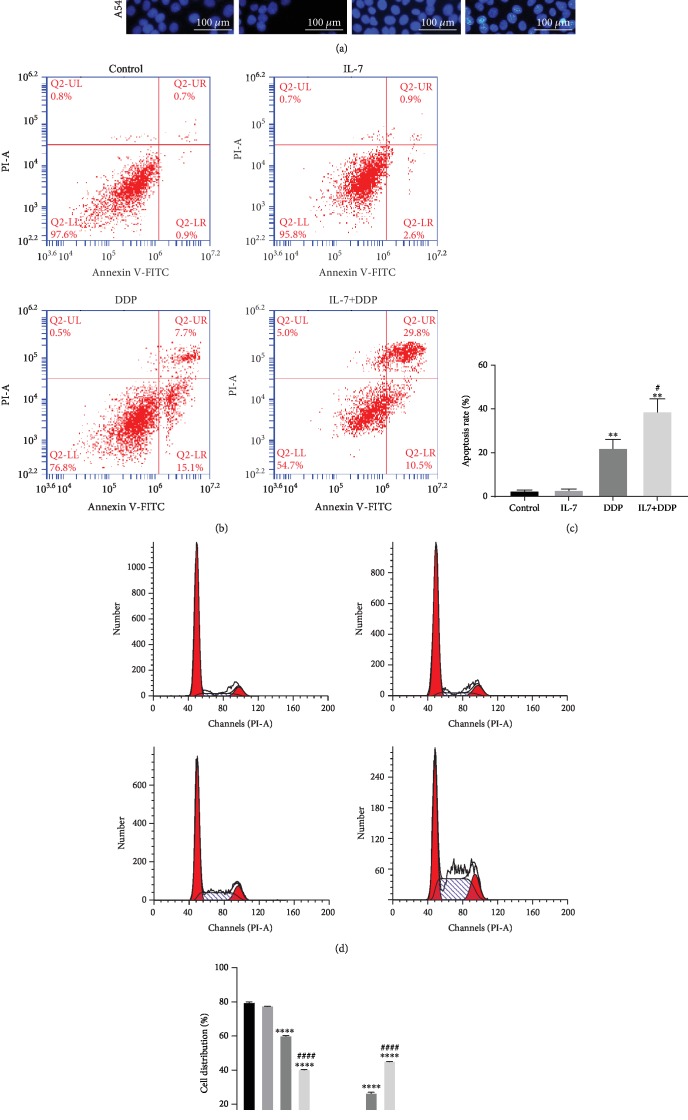
IL-7/DDP treatment promoted DDP-induced apoptosis and cell cycle arrest in A549/DDP. After the indicated treatment for 72 h in A549 and A549/DDP cells, the cell apoptosis was determined by Hoechst assay (a) and Annexin V-FITC/PI staining assay (b, c). In addition, the cell cycle of A549/DDP cells was analyzed by flow cytometry (d, e). ^∗∗^*p* < 0.01 and ^∗∗∗∗^*p* < 0.0001, as compared with the control; ^#^*p* < 0.05 and ^####^*p* < 0.0001 as compared with the DDP group. Data are presented in terms of mean ± SD.

IL-7-induced restoration of sensitivity to DDP is often found to be accompanied by the change of apoptosis and cell cycle. The IL-7-sensitizing effect to DDP was compared with A549/DDP and A549 cells. Apoptotic cells were observed in A549 cells when treated with DDP, whereas none were observed in A549/DDP cells treated with DDP in the Hoechst assay (Figure 2(a)). Moreover, IL-7 treatment sensitized the A549/DDP cells to DDP and was found to significantly induce cell apoptosis, as found with the Annexin V-FITC/PI staining apoptosis assay (Figures 2(b) and 2(c)). The cell cycle was also analyzed, and no inhibitory effect on the cell cycle was found when treated only with IL-7, but the percentage of cells in the S phase was found to be significantly higher after DDP treatment in A549/DDP cells (Figures 2(d) and 2(e)). Overall, IL-7 could restore the inhibitory effect of DDP on the tumor characteristics of the DDP-resistant cell line A549/DDP.

### 3.3. IL-7/DDP Treatment Inhibited PI3K Signals and ABCG2 Expression

**Figure 3:**
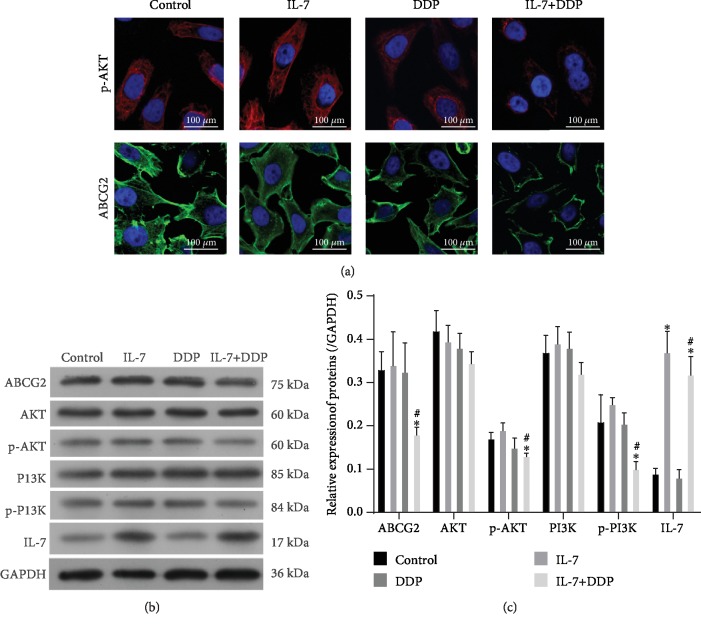
IL-7/DDP treatment inhibited the PI3K signal and the expression of ABCG2. After exposure to IL-7, DDR, or their combination for 72 h, the A549/DDP cells were harvested. In the cells, the levels of phosphorylated AKT and ABCG2 were examined by immunofluorescence assay (a). With Western blot analysis, the expressions of ABCG2, AKT, p-AKT, PI3K, p-PI3K, and IL-7 were examined (b) and then quantified (c). ^∗^*p* < 0.05, as compared with the control; ^#^*p* < 0.05, as compared with the DDP group. Data are presented in terms of mean ± SD.

As the PI3K/AKT pathway is activated by IL-7/IL 7R signals, these signals were further evaluated during the chemotherapy against A549/DDP cells. Phosphorylation of AKT and the expression of the multidrug-resistant transporter ABCG2 were inhibited by IL-7/DDP treatment, but not by IL-7 alone as indicated by the immunofluorescence assay (Figure 3(a)). The inhibition of the PI3K/AKT signal and ABCG2 expression by IL-7/DDP treatment was also validated by Western blot. As shown in Figures 3(b) and 3(c), their levels in the DDP group were similar to those in the control but were found to be significantly inhibited in the IL-7/DDP group. These data demonstrated that additional treatment with IL-7 sensitized PI3K/Akt signaling and ABCG2 expression to DDP. To get an impression of the specificity of IL-7's effects, IL-2 and IL-8 were used in the cisplatin sensitivity assays. As shown in Supplementary [Supplementary-material supplementary-material-1], the expression level of proteins was not found to change in other groups when compared with the control group. Moreover, IL-7 not IL-2 in combination with DDP could significantly reduce cell viability of A549/DDP cells when compared with the DDP treatment alone, as shown by the CCK-8 assay (Supplementary [Supplementary-material supplementary-material-1]). These data indicated that IL-2 did not affect the sensitivity of DDP.

### 3.4. PI3K Activating or Blocking IL-7 Signal Reversed IL-7-Induced Sensitivity of Resistant Cells to DDP

**Figure 4:**
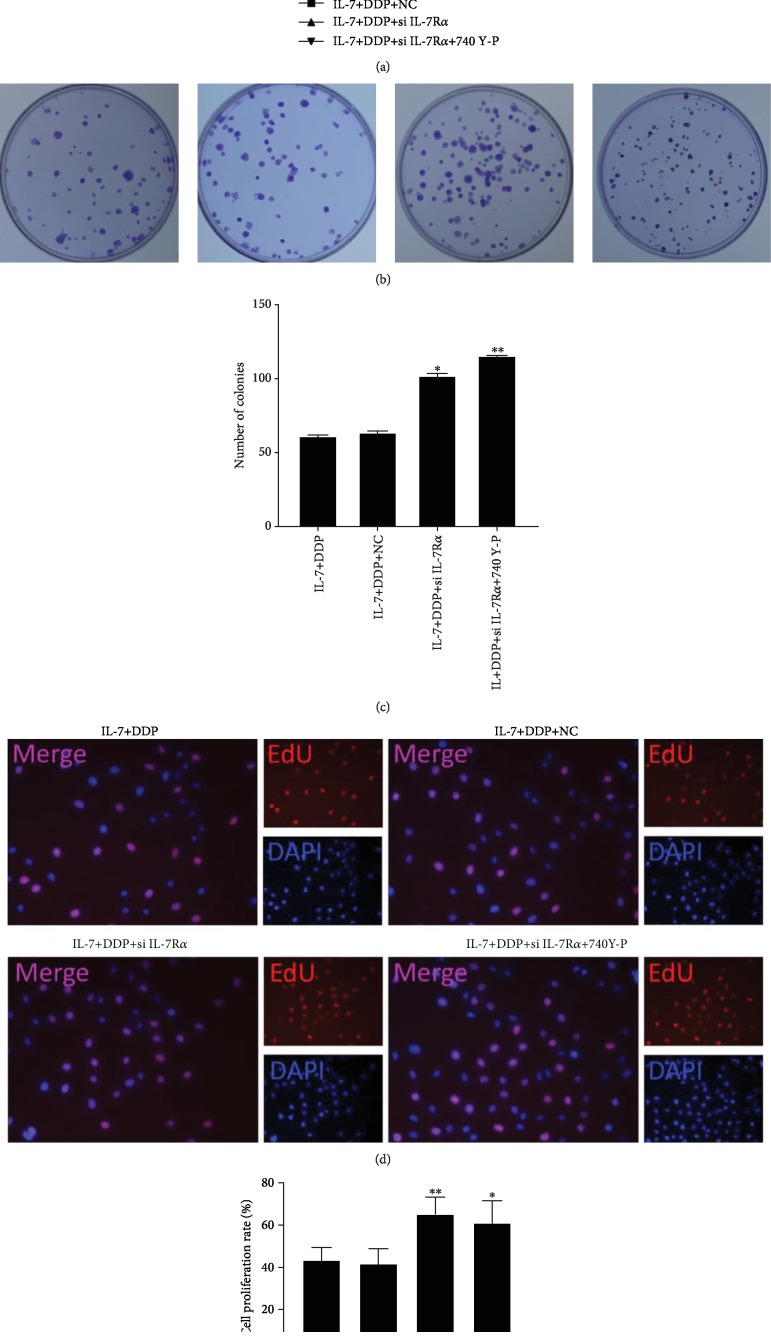
Both IL-7R knockdown and PI3K activation suppressed IL-7-ehanced cell proliferation by DDP. After being pretreated with siRNA against IL-7R*α* (si IL-7R*α*), 740 Y-P (10 nM), or control, the A549/DDR cells were exposed to IL-7 (2 ng/mL) and DDP (0.5 *μ*g/mL). At 24 h, 48 h, and 72 h after the combinational treatment, the cell viabilities were determined by CCK-8 assays (a). At 48 h after the combinational treatment, the proliferation was evaluated by colony formation assay (b, c) and by EdU staining (d, e). NC means cells were pretreated with negative control siRNA. ^∗^*p* < 0.05 and ^∗∗^*p* < 0.01, as compared with the cells pretreated with the control. Data are presented in terms of means ± SD.

To investigate the role of IL-7 and PI3K/AKT signals in the IL-7-induced resensitization of the resistant cells to DDP, they were inhibited and activated by siRNA against IL-7R*α* (si IL-7R*α*) and 740 Y-P, respectively. The knockdown efficiencies were explored via flow cytometry assays, and satisfactory results were obtained (Supplementary Figures [Supplementary-material supplementary-material-1]). As shown in Figures 4(a)–4(e), both si IL-7R*α* and 740 Y-P were significantly found to increase cell viabilities, colony numbers, and EdU-staining cells in A549/DDP cells treated with IL-7 and DDP, demonstrating that both IL-7 and PI3K signals play important roles in the resensitized effect of IL-7 on the resistant cells.

**Figure 5:**
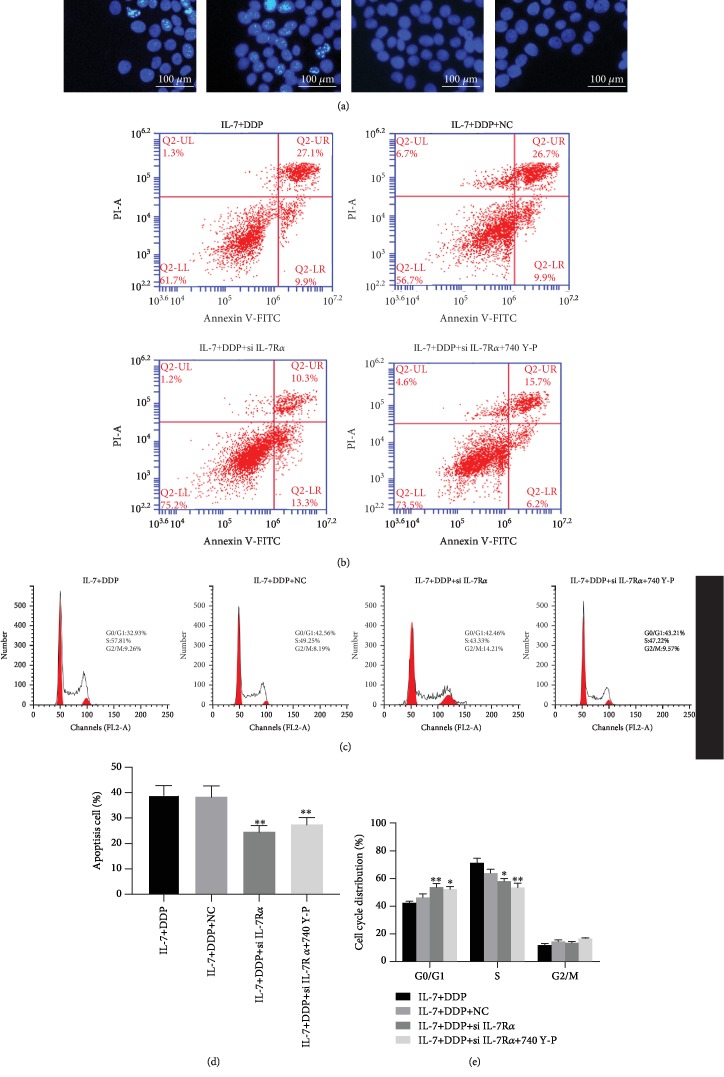
Both IL-7R knockdown and PI3K activation inhibited the synergetic effect by IL-7 on the apoptosis and cell cycle arrest induced by DDP. After being pretreated with si IL-7R or NC transfection, or 740 Y-P (10 nM), the A549/DDR cells were exposed to IL-7 (2 ng/mL) and DDP (0.5 *μ*g/mL). At 48 h after the combinational treatment, the cell apoptosis was determined by Hoechst staining (a) and flow cytometry (b, d) and the cell cycle was also examined by flow cytometry (c, e). NC means cells were pretreated with negative control siRNA. ^∗^*p* < 0.05 and ^∗∗^*p* < 0.01, as compared with the cells pretreated with the control. Data are presented in terms of mean ± SD.

Hoechst staining and flow cytometry indicated that both si IL-7R*α* and 740 Y-P reduced the apoptosis in the combination-treated A549/DDP cells (Figures 5(a), 5(b), and 5(d)). Cell cycle analysis also showed the percentage of A549/DDP cells in the S phase induced by IL-7+DDP treatment to be reduced posttreatment with si IL-7R*α* and 740 Y-P (Figures 5(c) and 5(e)). These results confirmed the crucial role of the IL-7/PI3K signal in IL-7/DDP-induced sensitivity of the A549/DDP cell line.

### 3.5. Si IL-7R*α* or 740 Y-P Treatment Rescued the Expression of ABCG2 after IL-7/DDP Treatment

**Figure 6:**
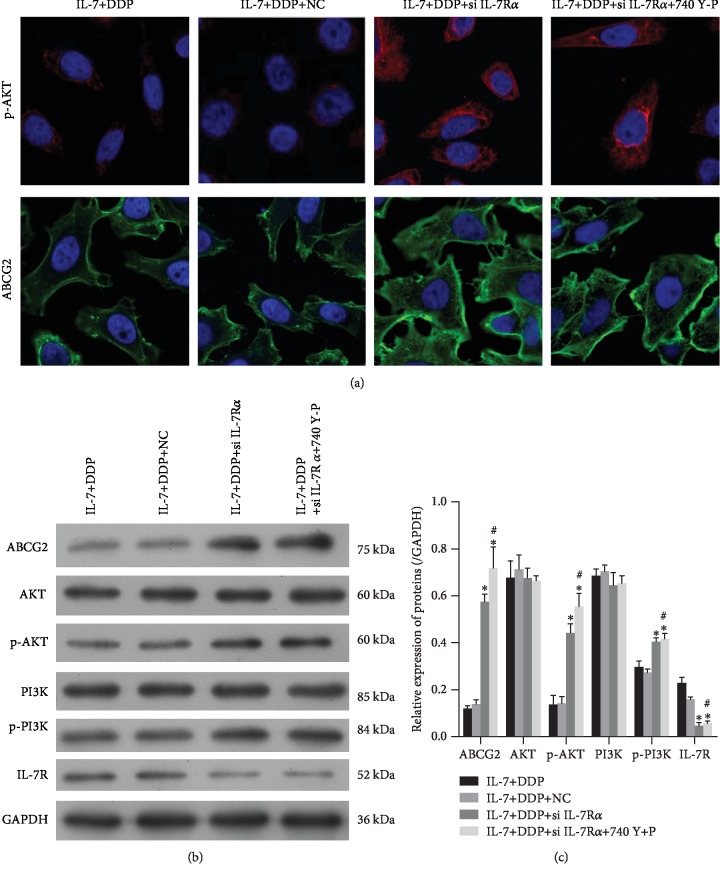
Both IL-7R knockdown and PI3K activation inhibited the synergetic effect by IL-7 on decreased expression of ABCG2 by DDP. After being pretreated with si IL-7R, 740 Y-P, or control, the A549/DDR cells were exposed to IL-7 (2 ng/mL) and DDP (0.5 *μ*g/mL). At 48 h, the cells were harvested for examinations on the expressions of p-AKT and ABCG2 by immunofluorescence (a) and with Western blot, along with PI3K, p-PI3K, and IL-7R (b, c). NC means cells were pretreated with negative control siRNA. ^∗^*p* < 0.05, as compared with the IL-7+DDP+NC group; ^#^*p* < 0.05, as compared with the IL-7+DDP+si IL-7R group. Data are presented in terms of mean ± SD.

The impact of the IL-7/PI3K signal on the expression of ABCG2 was assessed. The phosphorylation of AKT and expression of ABCG2 were significantly observed to be higher in IL-7/DDP-treated A549/DDP cells by using si IL-7R*α* or 740 Y-P (Figure 6(a)) as indicated by the immunofluorescence assay. This was also established by the Western blot (Figures 6(b) and 6(c)). Thus, IL-7/DDP treatment can be said to suppress the expression of ABCG2 through the IL-7/PI3K/AKT signal to sensitize A549/DDP cells.

### 3.6. The Combination of IL-7 with DDP Efficiently Upregulates DDP Sensitivity In Vivo

**Figure 7:**
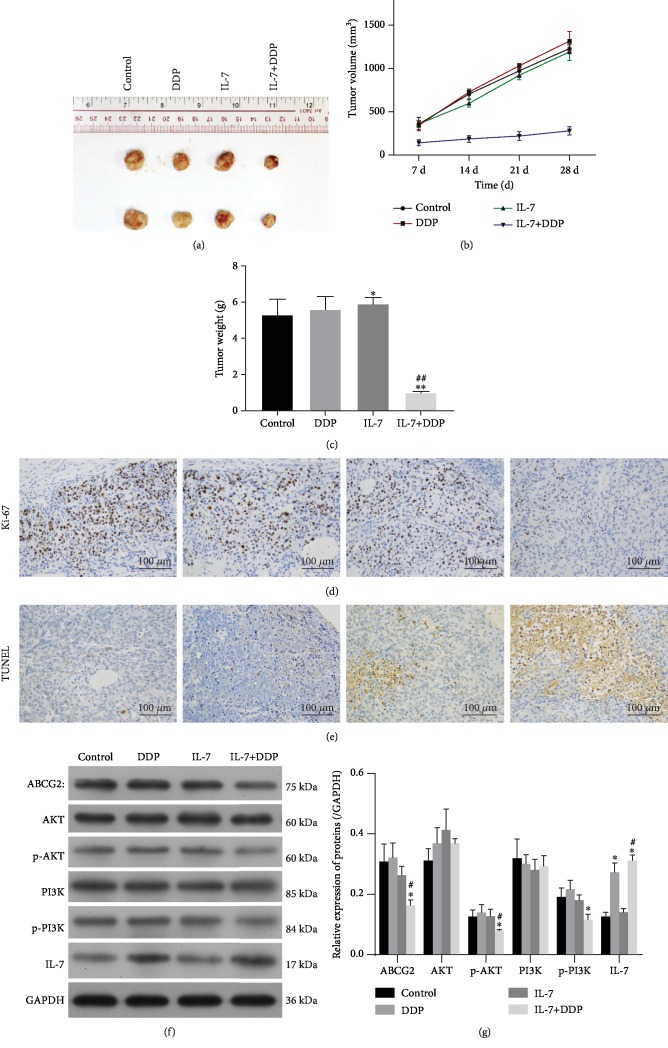
Treatment of IL-7 in vivo restored the DDP sensitivity of tumors in mice. The xenograft tumor resistant to DDP was established by injection of A549/DDP into the nude mice. Subsequently, the mice were treated with IL-7 (2 *μ*g/injection, twice a week) and DDP (5 mg/kg, twice a week). After 28-day treatment, the mice were sacrificed and the tumors were collected for examinations on the volume (a, b), the weight (c), the cellular expression of Ki-76 by IHC (d), the cell apoptosis by TUNEL assay (e), and the expressions of ABCG2, AKT, p-AKT, PI3K, p-PI3K, and IL-7 by Western blot (f, g). ^∗^*p* < 0.05 and ^∗∗^*p* < 0.01, as compared with the DDP group; ^#^*p* < 0.05, as compared with the DDP group. Data are presented in terms of mean ± SD.

The antitumor efficacy of IL-7 in combination with DDP was analyzed in vivo. The xenograft model of the DDP-resistant human lung cancer cell line A549/DDP was established, and nude mice were treated with IL-7 (2 *μ*g/injection, twice a week) and DDP (5 mg/kg, twice a week). Results indicated that IL-7 treatment alone was not observed to significantly affect tumor growth, but the combination of IL-7 and DDP was found to inhibit tumor growth resulting in reduced tumor volume in mice with A549/DDP (Figures 7(a)–7(c)). These results were found consistent with the decreased expression of proliferation index Ki-67 (Figure 7(d)) and the upregulated apoptosis (Figure 7(e)) in tumor tissues. PI3K signals and the expression of ABCG2 were also analyzed in the tumor samples. The treatment of IL-7/DDP significantly downregulated the activation of the PI3K/AKT signal and expression of ABCG2 in vivo (Figures 7(f) and 7(g)), which counteracted with DDP-induced chemotherapy resistance in vivo.

## 4. Discussion

The majority of NSCLC patients often present with locally advanced or metastatic disease at diagnosis, thus rendering NSCLC as one of the most challenging malignancies to be treated [19]. Platinum-based chemotherapy in combination with or without maintenance therapy and subsequent second-line cytotoxic chemotherapy are standard treatments for patients with advanced NSCLC, but the 5-year survival has not been found to improve and remains only 20% because of the side effects and therapy resistance [20]. We applied an IL-7-dependent therapy to overcome the chemotherapy resistance in NSCLC. IL-7 was observed to restore the sensitivity of DDP in the A549/DDP cell line via the suppressing PI3K/AKT pathway, although IL-7 alone had no effect on tumor eradication.

Increasing evidence has demonstrated that cytokines could be increasingly applied in cancer immunotherapy. For instance, IL-2 has been widely used for the treatment of patients with metastatic melanoma [21]. IL-7 has been largely reported to play a key role in the adaptive immune system, it being a nonhematopoietic cell-derived cytokine. Many researchers have focused on its potential antitumor effects on tumors in addition to its immunological function, mainly including glioma, prostate cancer, and glioblastoma. IL-7 is often utilized to enhance the efficacy of tumor regression. For instance, there is observed a relapse-free survival and the inhibition of pulmonary metastasis nodules by treating with IL-7 and IL-15 after radiofrequency thermal ablation (RFA) in mammary carcinoma [12]. Miller et al. have also found that intratumoral injection with adenoviral IL-7-transduced dendritic cells could cause complete tumor regression in a murine lung cancer model [22]. Given its role in immunity and the pathogenesis of neoplasia, it was opted to explore IL-7's role in the multidrug resistance of DDP in NSCLCs.

Actually, treatment with IL-7 *in vitro* and *in vivo* showed inconsistent function in diverse contexts. Insufficient IL-7 limits the survival and the persistence of memory CD8^+^ T cells. Yet administration of IL-7 enhanced the number of memory CD8^+^ T cells in the contraction phase of the response during the mouse T cell response to lymphocytic choriomeningitis virus [23]. Nevertheless, the functions of promoting survival and proliferation of T cells were also responsible for hematological malignancies including leukemia and lymphoma. Barata et al. found that IL-7 induced PI3K-dependent phosphorylation of AKT resulting in Bcl-2 upregulation, p27^kip1^ downregulation, and Rb hyperphosphorylation, eventually leading to the growth and proliferation of T-ALL cells [24]. Phosphorylation of PI3K was not observed to be influenced by either IL-7 or DDP treatment in our experiments. However, the combination of IL-7 and DDP was significantly found to suppress p-PI3K, and PI3K activated by 740 Y-P could reverse the influence of IL-7 on cisplatin sensitivity. This situation may reflect different timelines and action time frames of two PI3K signals under diverse circumstances, and the specific mechanism of PI3K observed in this study needs to be further investigated. As IL-7 can augment the function of tumor-reactive CD8^+^ T cells, accumulating research has demonstrated recombinant IL-7 to be an adjuvant for adoptive immunotherapy. Ding et al. found endogenous IL-7 to enhance donor CD4^+^ effector T cell expansion and persistence after lymphodepleting chemotherapy, improving the therapeutic outcome in a mouse lymphoma model [25]. But in a solid tumor, the IL-7/IL-7R axis promoted prostate cancer cell invasion and migration by activating the AKT/NF-*κ*B pathway and increasing MMP-3 and MMP-7 expression [8]. A relative low dose and frequency of IL-7 treatment in our experiments exhibited no effects on the xenograft models of human lung cancer cells, but some studies reported that once the IL-7 treatment was performed daily (5 *μ*g/injection) in cases of lung cancer, the tumor regression was observed to be induced efficiently [26]. Conversely, Wang et al. found that IL-7 (20 *μ*g/mL/kg) administered every two days to mice with lung cancer significantly stimulated the development of tumor by increasing the expression of cyclin D1 and phosphorylation of c-Fos/c-Jun signals [27]. Therefore, the inconsistent functioning of IL-7 in lung cancer is attributable to the discrepancy of dosage, administration, and combination schedule.

Despite the discrepant functioning of IL-7 in various models, the combinational therapies of IL-7 with other drugs have shown enhanced efficiency of such therapies. Infection of *Haemophilus influenzae* (NTHi) results in IL-12 and IL-7 synergistically controlling granzyme B by upregulating the IL-12 receptor in lung CD4^+^ and CD8^+^ T cells, which are used for increasing antibacterial response [28]. The combination of IL-21 and IL-7 possesses potent antitumor immune activity in whole-cell vaccines with enhanced infiltration of effector T cells [29]. Besides, in vivo administration of IL-7 in combination with oxaliplatin was found to remarkably inhibit the growth of tumors in lung and abdominal metastasis models of colon cancer by reactivating the immune system [30]. However, IL-7 was found to overcome DDP resistance in the chemotherapy of NSCLC by downregulating MDR genes independent of the immune system. Ding et al. similarly found an immune agonist poly(I:C) to inhibit drug efflux and upregulate DDP-induced cytotoxicity, which was independent of the immune system [31].

Interestingly, we proved that DDP/DDP+IL-7 treatment significantly increased the accumulation of cells in the S phase. We confirmed this phenomenon via the results obtained from previous studies. For instance, Tan et al. proved that pterostilbene could induce the cell cycle arrest at the S phase via upregulating the caspase-3, caspase-8, caspase-9, and Bax protein expression and downregulating the Bcl-2 expression [32]. Meanwhile, they also proved that pterostilbene suppressed cyclins A and E which regulate the progression to the G2/M phase and promoted the p21 and p27 expression which functioned as the CDK inhibitors. Therefore, we speculated that DDP/DDP+IL-7 treatment might also induce the cell cycle arrest at the S phase via regulating the apoptosis-related caspase and Bcl-2 family proteins. However, we need to further explore this hypothesis.

To conclude, we established through our experiments that IL-7 treatment is conducive to overcoming DDP resistance in NSCLC, which was involved in the inhibition of the PI3K/AKT pathway and multidrug resistance.

## Data Availability

Data is available on request. The data used to support the findings of this study were supplied by Dr. Bin Ke under license and so cannot be made freely available. Requests for access to these data should be made to Dr. Bin Ke, jackhorn@163.com.

## Supplementary Materials

Supplementary Materials Expression of IL-7R detected by flow cytometry (A and B) after being transfected with IL-7R alpha siRNA and the corresponding negative control for 48 h. The A549/DDR cells were exposed to IL-2 (2 ng/mL) or IL-8 (2 ng/mL) with or without DDP (0.5 *μ*g/mL). At 48 h, the cells were harvested for examinations on the expressions of p-AKT and ABCG2 by Western blot along with PI3K and p-PI3K (C). The influences of different concentrations of IL-7 on the proliferation of A549 and A549/DDP cells were explored via CCK-8 assays (D). CCK-8 assay was performed to detect the effect of IL-7 and IL-2 on cell viability of DDP-treated A549/DDP cells (E). ^∗^*p* < 0.05, ^∗∗^*p* < 0.01, ^∗∗∗^*p* < 0.001, and ^∗∗∗∗^*p* < 0.0001, as compared with the control group. ^####^*p* < 0.0001, as compared with the DDP group. Data are presented in terms of mean ± SD.

## References

[1] Galanski M., Jakupec M. A., Keppler B. K. (2005). Update of the preclinical situation of anticancer platinum complexes: novel design strategies and innovative analytical approaches. *Current Medicinal Chemistry*.

[2] Ghosh S. (2019). Cisplatin: the first metal based anticancer drug. *Bioorganic Chemistry*.

[3] Chaudhary K. R., Yan S. X., Heilbroner S. P. (2019). Effects of *β*-Adrenergic antagonists on chemoradiation therapy for locally advanced non-small cell lung cancer. *Journal of Clinical Medicine*.

[4] Vacchelli E., Ma Y., Baracco E. E., Zitvogel L., Kroemer G. (2016). Yet another pattern recognition receptor involved in the chemotherapy-induced anticancer immune response: formyl peptide receptor-1. *Oncoimmunology*.

[5] Corrales L., Matson V., Flood B., Spranger S., Gajewski T. F. (2017). Innate immune signaling and regulation in cancer immunotherapy. *Cell Research*.

[6] Belarif L., Mary C., Jacquemont L. (2018). IL-7 receptor blockade blunts antigen-specific memory T cell responses and chronic inflammation in primates. *Nature Communications*.

[7] Mackall C. L., Fry T. J., Gress R. E. (2011). Harnessing the biology of IL-7 for therapeutic application. *Nature Reviews. Immunology*.

[8] Qu H., Zou Z., Pan Z. (2016). IL-7/IL-7 receptor axis stimulates prostate cancer cell invasion and migration via AKT/NF-*κ*B pathway. *International Immunopharmacology*.

[9] Park S. L., Lee E. J., Kim W. J., Moon S. K. (2014). p27KIP1 is involved in ERK1/2-mediated MMP-9 expression via the activation of NF-*κ*B binding in the IL-7-induced migration and invasion of 5637 cells. *International Journal of Oncology*.

[10] Lin J., Zhu Z., Xiao H. (2017). The role of IL-7 in immunity and cancer. *Anticancer Research*.

[11] Sharma S., Batra R. K., Yang S. C. (2003). Interleukin-7 gene-modified dendritic cells reduce pulmonary tumor burden in spontaneous murine bronchoalveolar cell carcinoma. *Human Gene Therapy*.

[12] Habibi M., Kmieciak M., Graham L., Morales J. K., Bear H. D., Manjili M. H. (2009). Radiofrequency thermal ablation of breast tumors combined with intralesional administration of IL-7 and IL-15 augments anti-tumor immune responses and inhibits tumor development and metastasis. *Breast Cancer Research and Treatment*.

[13] Kim Y. K., Lee S. S., Jeong S. H. (2014). OCT-1, ABCB1, and ABCG2 expression in imatinib-resistant chronic myeloid leukemia treated with dasatinib or nilotinib. *Chonnam Medical Journal*.

[14] Zhang W., Chen Z., Chen L. (2017). ABCG2-overexpressing H460/MX20 cell xenografts in athymic nude mice maintained original biochemical and cytological characteristics. *Scientific Reports*.

[15] Li X., Zou Z., Tang J. (2019). NOS1 upregulates ABCG2 expression contributing to DDP chemoresistance in ovarian cancer cells. *Oncology Letters*.

[16] Vesel M., Rapp J., Feller D. (2017). ABCB1 and ABCG2 drug transporters are differentially expressed in non-small cell lung cancers (NSCLC) and expression is modified by cisplatin treatment via altered Wnt signaling. *Respiratory Research*.

[17] Guo J., Jin D., Wu Y. (2018). The miR 495-UBE2C-ABCG2/ERCC1 axis reverses cisplatin resistance by downregulating drug resistance genes in cisplatin-resistant non-small cell lung cancer cells. *eBioMedicine*.

[18] Ding L., Ren J., Zhang D. (2018). A novel stromal lncRNA signature reprograms fibroblasts to promote the growth of oral squamous cell carcinoma via lncRNA-CAF/interleukin-33. *Carcinogenesis*.

[19] Langer C. J., Obasaju C., Bunn P. (2016). Incremental innovation and progress in advanced squamous cell lung cancer: current status and future impact of treatment. *Journal of Thoracic Oncology*.

[20] Dempke W. C., Sellmann L., Fenchel K., Edvardsen K. (2015). Immunotherapies for NSCLC: are we cutting the Gordian helix?. *Anticancer Research*.

[21] Rosenberg S. A. (2014). IL-2: the first effective immunotherapy for human cancer. *Journal of Immunology*.

[22] Miller P. W. S. S., Stolina M., Butterfield L. H. (2000). Intratumoral administration of adenoviral interleukin 7 gene-modified dendritic cells augments specific antitumor immunity and achieves tumor eradication. *Human Gene Therapy*.

[23] Pellegrini M., Calzascia T., Toe J. G. (2011). IL-7 engages multiple mechanisms to overcome chronic viral infection and limit organ pathology. *Cell*.

[24] Barata J. T., Silva A., Brandao J. G., Nadler L. M., Cardoso A. A., Boussiotis V. A. (2004). Activation of PI3K is indispensable for interleukin 7-mediated viability, proliferation, glucose use, and growth of T cell acute lymphoblastic leukemia cells. *The Journal of Experimental Medicine*.

[25] Ding Z. C., Habtetsion T., Cao Y. (2017). Adjuvant IL-7 potentiates adoptive T cell therapy by amplifying and sustaining polyfunctional antitumor CD4+ T cells. *Scientific Reports*.

[26] Andersson A., Yang S. C., Huang M. (2009). IL-7 promotes CXCR3 ligand-dependent T cell antitumor reactivity in lung cancer. *Journal of Immunology*.

[27] Ming J., Jiang G., Zhang Q., Qiu X., Wang E. (2012). Interleukin-7 up-regulates cyclin D1 via activator protein-1 to promote proliferation of cell in lung cancer. *Cancer Immunology, Immunotherapy*.

[28] Wallington J. C., Williams A. P., Staples K. J., Wilkinson T. M. A. (2017). IL-12 and IL-7 synergize to control mucosal-associated invariant T-cell cytotoxic responses to bacterial infection. *The Journal of Allergy and Clinical Immunology*.

[29] Gu Y. Z., Fan C. W., Lu R. (2016). Forced co-expression of IL-21 and IL-7 in whole-cell cancer vaccines promotes antitumor immunity. *Scientific Reports*.

[30] Gou H. F., Huang J., Shi H. S., Chen X. C., Wang Y. S. (2014). Chemo-immunotherapy with oxaliplatin and interleukin-7 inhibits colon cancer metastasis in mice. *PLoS One*.

[31] Crawley A. M., Vranjkovic A., Faller E. (2014). Jak/STAT and PI3K signaling pathways have both common and distinct roles in IL-7-mediated activities in human CD8^+^ T cells. *Journal of Leukocyte Biology*.

[32] Tan K. T. C. P., Li S., Ke T. M., Lin S. H., Yang C. C. (2019). Pterostilbene inhibits lung squamous cell carcinoma growth *in vitro* and *in vivo* by inducing S phase arrest and apoptosis. *Oncology Letters*.

