# Supplementary material for: Interleukin-7 Resensitizes Non-Small-Cell Lung Cancer to Cisplatin via Inhibition of ABCG2
Source: Mediators Inflamm. 2019 Dec 14;2019:7241418. doi: 10.1155/2019/7241418 (PMC6931030; doi:10.1155/2019/7241418)
Supplement: Supplementary Materials — Expression of IL-7R detected by flow cytometry (A and B) after being transfected with IL-7R alpha siRNA and the corresponding negative control for 48 h. The A549/DDR cells were exposed to IL-2 (2 ng/mL) or IL-8 (2 ng/mL) with or without DDP (0.5 μg/mL). At 48 h, the cells were harvested for examinations on the expressions of p-AKT and ABCG2 by Western blot along with PI3K and p-PI3K (C). The influences of different concentrations of IL-7 on the proliferation of A549 and A549/DDP cells were explored via CCK-8 assays (D). CCK-8 assay was performed to detect the effect of IL-7 and IL-2 on cell viability of DDP-treated A549/DDP cells (E). ∗p < 0.05, ∗∗p < 0.01, ∗∗∗p < 0.001, and ∗∗∗∗p < 0.0001, as compared with the control group. ####p < 0.0001, as compared with the DDP group. Data are presented in terms of mean ± SD. [file 7241418.f1.pdf]

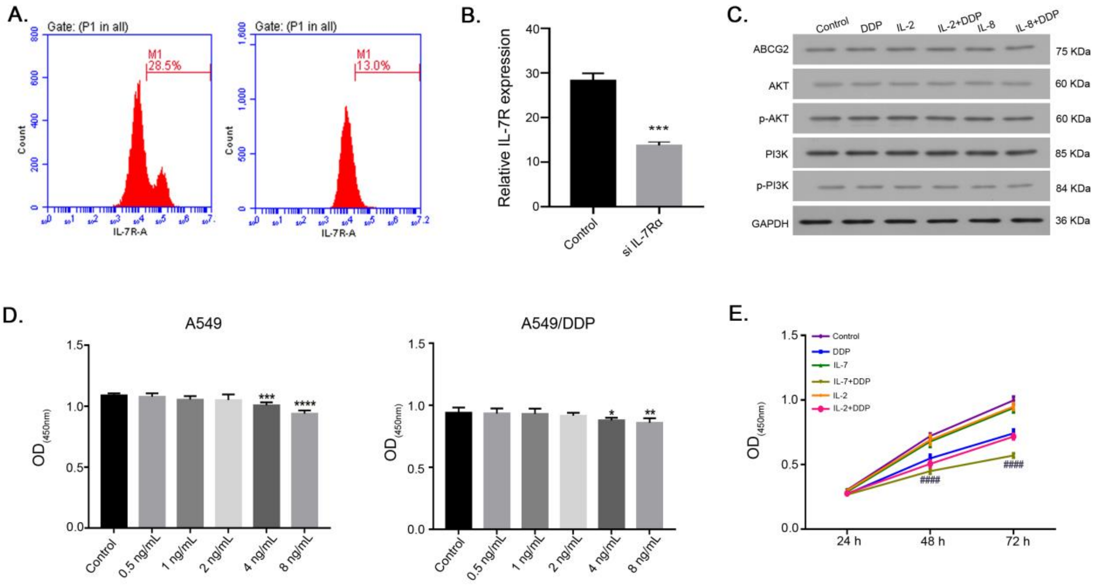

Supplementary Figure. Expression of IL-7R detected by flow cytometry (A and B) after transfected with IL-7R alpha siRNA and the corresponding negative control for 48 h. The A549/DDR cells were exposed to IL-2 (2 ng/mL) or IL-8 (2 ng/mL) with or without DDP (0.5 μg/mL). At 48 h, the cells were harvested for examinations on the expressions of p-AKT and ABCG2 by Western blot along with PI3K and p-PI3K (C). The influences of different concentrations of IL-7 on the proliferation of A549 and A549/DDP cells were explored via CCK-8 assays (D). CCK-8 asaay was performed to detected the effect of IL-7 and IL-2 on cell viability of DDP-treated A549/DDP cells (E). *$p < 0.05$, **$p < 0.01$, ***$p < 0.001$, ****$p < 0.0001$, as compared with the control group. ####$p < 0.0001$, as compared with the DDP group. Data are presented in terms of mean ± SD.
